# Kawasaki disease complicated with shock syndrome, macrophage activation syndrome, and acute abdomen in children: Two case reports

**DOI:** 10.3389/fped.2023.1152242

**Published:** 2023-04-21

**Authors:** Cong Yi, Xiang She, Jia Chen

**Affiliations:** Department of Pediatrics, Mianyang Central Hospital, School of Medicine, University of Electronic Science and Technology of China, Mianyang, China

**Keywords:** kawasaki disease, kawasaki disease shock syndrome, macrophage activation syndrome, acute abdomen, case report

## Abstract

**Background:**

Kawasaki disease (KD) is an acute systemic vasculitis that can involve multiple organs. Few reports have been published about KD patients presenting with multiple complications such as acute abdomen, KD shock syndrome (KDSS), and macrophage activation syndrome (MAS).

**Case Description:**

We present the cases of two males (9 and 12 years old) diagnosed with KD accompanied by rare manifestations. Case 1 is a 9-year-old male treated for acute appendicitis, KDSS, and MAS. Case 2 is a 12-year-old male who presented with KDSS, MAS, and an ileal perforation. They were treated with intravenous immunoglobulin, aspirin, high-dose corticosteroids, vasoactive drugs, and symptomatic treatment, with good outcomes.

**Conclusions:**

Clinicians should be aware of the possibility of KD in the presence of fever and unusual manifestations, such as severe inflammatory indicators and acute abdomen that is nonresponsive to antibiotic therapy. Meanwhile, KD-related unusual complications should be recognized, such as KDSS and MAS.

## Introduction

Kawasaki disease (KD) is a vasculitis syndrome of small- and medium-sized vessels (1). High-dose intravenous immunoglobulin (IVIG) plus aspirin has been the standard treatment for KD for over four decades (2). KD shock syndrome (KDSS) is a rare and potentially fatal manifestation of KD. In 2009, Kanegaye et al. first formally defined KDSS as KD with systolic hypotension or shock, with an incidence of 1%–7% (3, 4). Macrophage activation syndrome (MAS), also known as secondary hemophagocytic lymphohistiocytosis (HLH), is another relatively infrequent but severe complication of KD, representing 1.1%–1.9% of all KD patients (5, 6). Abdominal symptoms are relatively common in KD as markers of severity; however, acute surgical abdomen is a rare presentation with a prevalence of 4.6% (7). Patients with KD complicated by KDSS, MAS, or abdominal manifestations are at higher risk for IVIG resistance and the development of coronary artery abnormalities (CAAs) (5, 8, 9). Diagnosing KD and its complications is difficult and usually delayed, resulting in more CAAs. Here, we report two cases of an extremely rare type of KD complicated by KDSS, MAS, and acute abdomen.

## Case presentations

### Case 1

A previously healthy 9-year-old male presented with high fever, followed by bilateral conjunctival injection and generalized maculopapular rashes in July 2020. On day 4 of the illness, he developed abdominal pain and watery diarrhea and was admitted to a local hospital. Laboratory studies showed procalcitonin (PCT) 38.03 µg/L, C-reactive protein (CRP) 58.9 mg/L, white blood cell (WBC) 7.3 × 10^9^/L (neutrophils 78.4%), hemoglobin (Hb) 139 g/L, platelet (PLT) count 101 × 10^9^/L, alanine aminotransferase (ALT) 106 U/L, aspartate aminotransferase (AST) 497 U/L, ferritin > 2000 ng/ml. Serology test and reverse transcriptase (RT)-PCR for severe acute respiratory syndrome coronavirus 2 (SARS-CoV-2) were both negative. And he had no exposure history. Abdominal ultrasonography showed a swollen appendix with fecalith and luminal effusion, and a small effusion in the peritoneal cavity. Echocardiography was normal. Initial diagnosis was sepsis and acute appendicitis; thus, he was treated with intravenous antibiotics and IVIG (0.7 g/kg), but with no significant improvement. The patient was then transferred to the pediatric intensive care unit (PICU) of our hospital.

Upon admission (on day 6 of illness), physical examination revealed generalized maculopapular rashes, bilateral conjunctival injection, cracked lips, bilateral cervical lymphadenopathy (1.5 × 1.5 cm), edema of hands and feet, whole abdomen tenderness, and mild muscle tension without marked rebound tenderness. There was no palpable splenomegaly. Laboratory examinations showed CRP 74.71 mg/L, PCT 95.84 µg/L, WBC 6.53 × 10^9^/L (neutrophils 87.8%), Hb 89 g/L, PLT 96 × 10^9^/L, pro-brain natriuretic peptide (pro-BNP) 3,932 pg/ml, ferritin > 40,000 ng/ml, ALT 118 U/l, AST 590 U/l, albumin 22.32 g/L. Erythrocyte sedimentation rate (ESR) was normal. Coronary artery dilatation was not observed. Abdominal computed tomography (CT) scan revealed acute appendicitis (diameter: 8 mm) with localized peritonitis. According to these above clinical and laboratory findings, he was diagnosed with typical KD combined with acute appendicitis simultaneously and was promptly treated with additional dose of IVIG (1.3 g/kg), oral aspirin (30 mg/kg/day), and intravenous vancomycin combined with meropenem.

**Figure 1:**
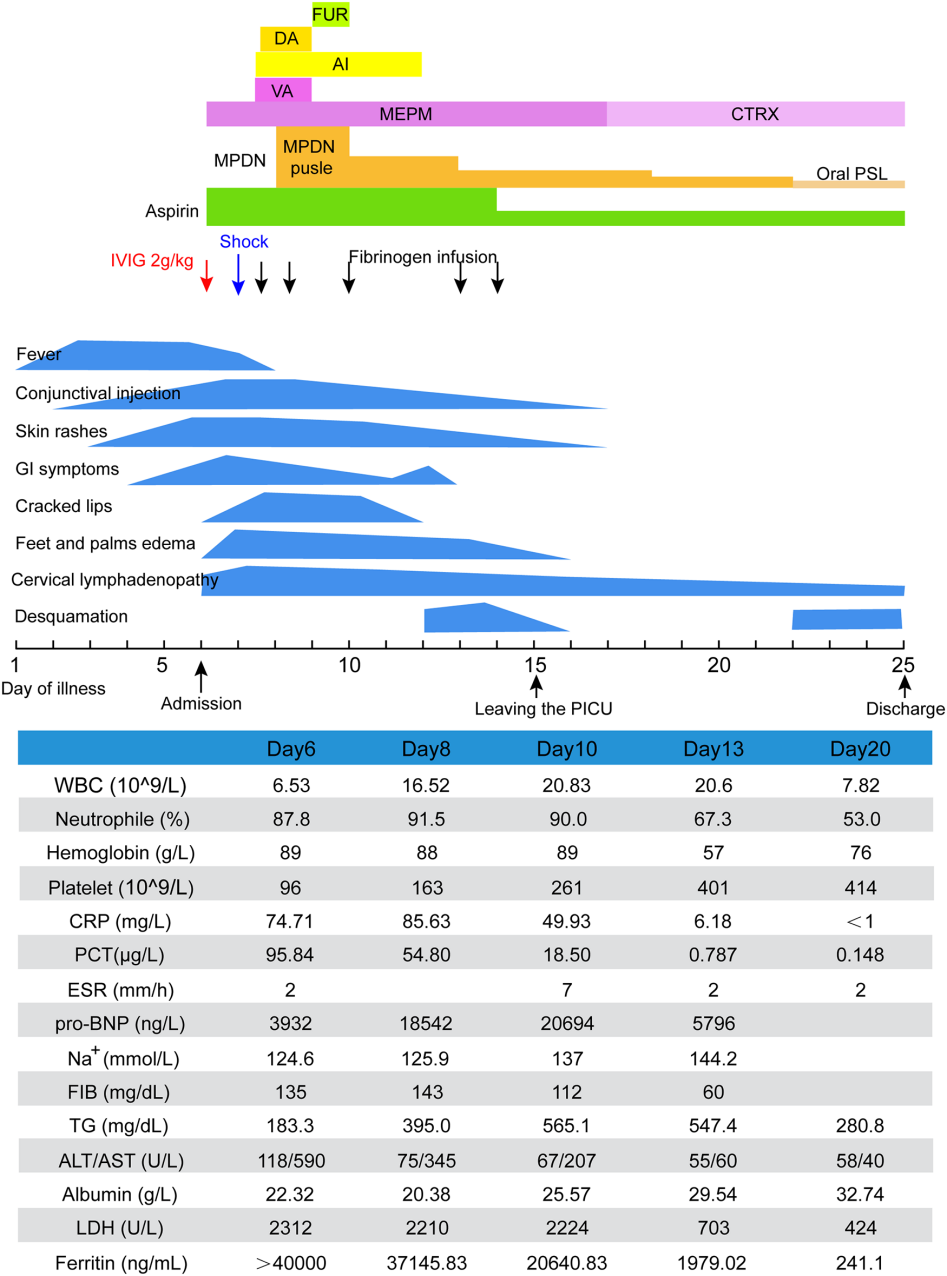
Clinical course of case 1. AI, albumin infusion; ALT, alanine transaminase; AST, aspartate aminotransferase; CRP, C-reactive protein; CTRX, ceftriaxone; DA, dopamine; ESR, erythrocyte sedimentation rate; FIB, fibrinogen; FUR, furosemide; IVIG, intravenous immunoglobulin; LDH, lactate dehydrogenase; MEPM, meropenem; MPDN, methylprednisolone; PCT, procalcitonin; pro-BNP, pro-brain natriuretic peptide; PSL, prednisolone; TG, triglycerides; VA, vancomycin; WBC, white blood cell.

On day 7, the patient suddenly presented with hypotension (75/30 mmHg), tachycardia (128 beats/min), and tachypnoea (36 breaths/min). Laboratory examinations revealed hyperferritinemia (37,145.83 ng/ml), thrombocytopenia (163 × 10^9^/L), hypofibrinogenemia (143 mg/dl), hypertriglyceridemia (395.0 mg/dl), and elevated AST 345 U/L and lactate dehydrogenase (LDH) (2,210 U/L). Bone marrow cytology did not detect the hemophagocytic phenomenon. Therefore, the patient was diagnosed as KD with concurrent KDSS and MAS. Low-dose dopamine was administered along with intravenous pulse methylprednisolone (MPDN; 25 mg/kg/day for three days), significantly improving clinical and biochemical abnormalities (Figure 1). On day 11, he became afebrile for two days, and MPDN was reduced to 4 mg/kg/day and tapered over 12 days, followed by oral prednisolone. On day 15, oral aspirin was switched to 5 mg/kg/day and the patient was transferred out of the PICU. On day 17, repeat echocardiography and abdominal ultrasonography were normal. On day 25 of the illness, he was discharged on aspirin (5 mg/kg/day) and tapering doses of oral prednisolone (initial dose 15 mg/day). The patient remained asymptomatic after two years of follow-up.

### Case 2

A 12-year-old previously healthy male presented with fever and a left neck mass with limited neck movement for two days in October 2020. On day 3 of the illness, he developed a maculopapular rash all over the body and was admitted to a local hospital. Laboratory investigations revealed neutrophilic leucocytosis and high CRP and PCT levels, with negative RT-PCR and serology test for SARS-CoV-2. He had no history of direct or indirect contact with COVID-19 patients. He was diagnosed with sepsis and was treated with intravenous antimicrobials. On day 5, bilateral non-purulent conjunctivitis was observed. Due to symptom persistence, the patient was transferred to the PICU of our hospital.

Upon admission at the PICU (on day 6 of illness), the patient developed a left neck mass, fissured lips, strawberry tongue, bilateral non-purulent conjunctivitis, generalized maculopapular rashes, and erythema without edema of the hands and feet. Splenomegaly was not detectable. Laboratory tests revealed marked leukocytosis (23.44 × 10^9^/L), anemia (Hb, 109 g/L), elevated CRP (219.79 mg/L) and PCT (1.71 µg/L), normal PLT (172 × 10^9^/L), with negative RT-PCR for SARS-CoV-2. Echocardiographic results were normal. Based on these findings, the patient's diagnosis of KD was confirmed. Hence, IVIG (2 g/kg) and aspirin (30 mg/kg/day) were administered immediately. He also received anti-infective treatment with piperacillin/tazobactam. As the fever persisted 36 h after IVIG treatment, a second dose of IVIG was administered, along with a small dose of intravenous MPDN (2 mg/kg/day), while aspirin was continued.

There was persistence of fever, severe hypotension (45/23 mmHg), generalized edema, tachycardia, oliguria, poor spirit, frequent vomiting, and tachypnoea with an oxygen saturation of approximately 90% through mask ventilation. Laboratory testing revealed hyperferritinemia (785.57 ng/ml), hypertriglyceridemia (311.8 mg/dl), hypofibrinogenemia (310 mg/dl), hyponatremia, hypokalemia, anemia (87 g/L), thrombocytopenia (106 × 10^9^/L), elevated pro-BNP (>70,000 ng/L) and creatinine (188.7 µmol/L), and reduced glomerular filtration rate (GFR, 16.7 ml/min/1.7m^2^). Echocardiography revealed mild right coronary dilation [left main coronary artery, LMCA 4.2 mm (+1.75 Z); right coronary artery, RCA 4.2 mm (+2.13 Z)]. Bone marrow aspirate and cerebrospinal fluid examination results were normal. Clinical deterioration was attributed to KDSS with MAS.

**Figure 2:**
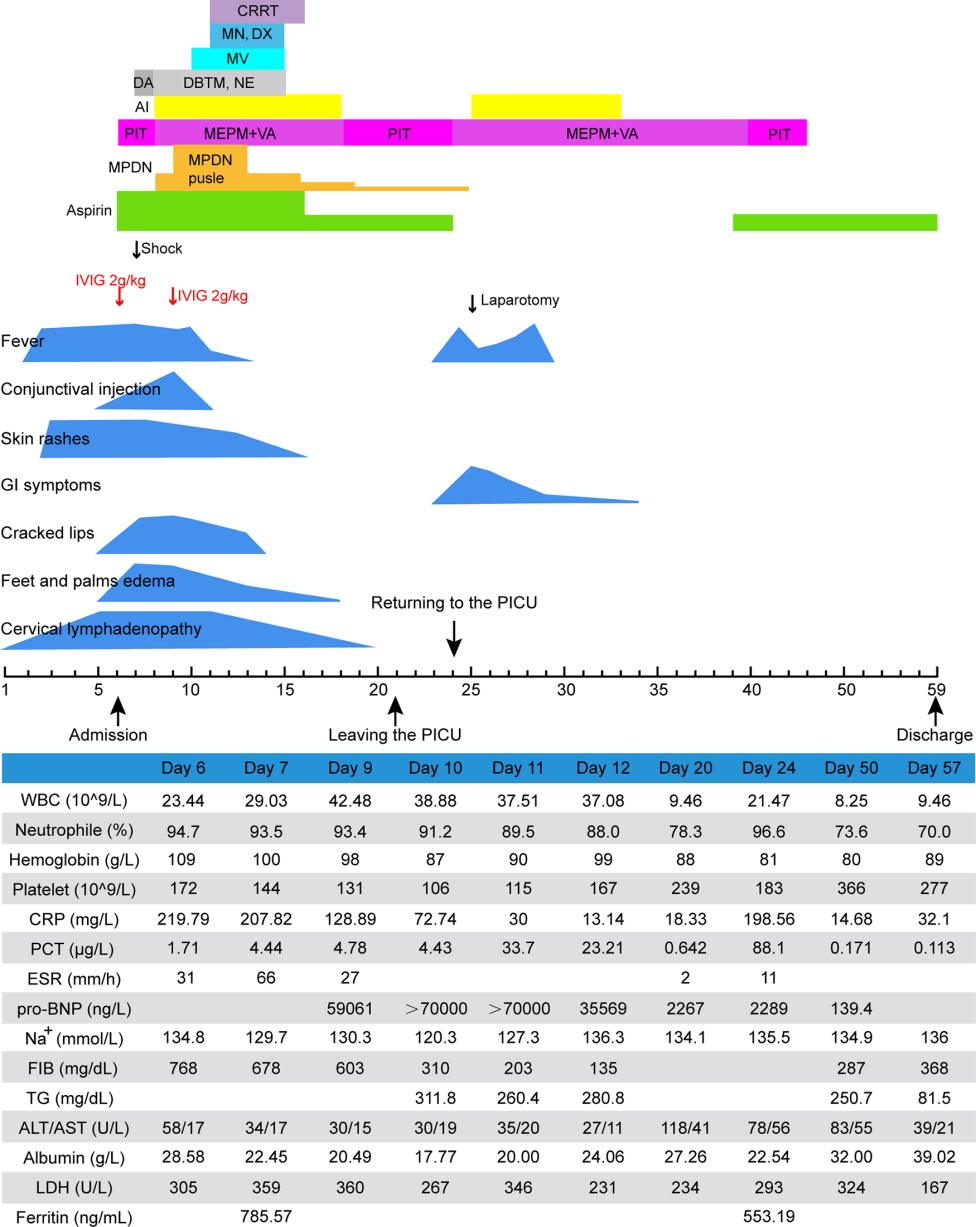
Clinical course of case 2. AI, albumin infusion; ALT, alanine transaminase; AST, aspartate aminotransferase; CRP, C-reactive protein; CRRT, continuous renal replacement therapy; DA, dopamine; DBTM, dobutamine; DX, digoxin; ESR, erythrocyte sedimentation rate; FIB, fibrinogen; IVIG, intravenous immunoglobulin; LDH, lactate dehydrogenase; MEPM, meropenem; MN, milrinone; MPDN, methylprednisolone; MV, mechanical ventilation; NE, norepinephrine; PCT, procalcitonin; PIT, piperacillin-tazobactam; pro-BNP, pro-brain natriuretic peptide; TG, triglycerides; VA, vancomycin; WBC, white blood cell.

After the patient was administered pulse intravenous MPDN (15 mg/kg/day for three days), low-dose maintenance therapy, vasoactive drugs, invasive mechanical ventilation, cardiac and diuretic treatment, continuous renal replacement therapy (CRRT), fluid resuscitation, antibiotic escalation to vancomycin and meropenem, correction of electrolyte disturbances, repeated intravenous human albumin infusion, and blood transfusion, the symptoms and laboratory test results gradually improved (Figure 2). On day 21, he was transferred out of the PICU. Unexpectedly, he developed recurrent fever and abdominal pain and distention after two days. Laboratory tests revealed elevated levels of inflammatory markers. Abdominal CT revealed free intraperitoneal air, suggesting gastrointestinal perforation. Emergency laparotomy revealed an ileal perforation, with subsequent resection of the necrotic intestine and ileostomy. Pathological examination revealed extensive necrosis with a massive infiltration of inflammatory cells. The patient was maintained on antibiotics and oral aspirin. His general condition gradually improved. Repeated echocardiography revealed normal coronary artery diameters [LMCA 3.1 mm (−0.33 Z); RCA 2.7 mm (−0.51 Z)]. He was discharged 53 days after the hospitalization. Ileostomy closure was performed two months later. The patient has remained well on follow-up for 1.9 years so far.

## Discussion

KD is a multisystem vasculitis of unknown etiology and pathogenesis. The diagnosis of KD is based on the presence of characteristic clinical manifestations, including prolonged fever (more than five days without other more reasonable explanation) and any four or more of the following five major symptoms: oral mucosa change, skin rashes, swollen palms or soles, cervical lymphadenitis, and non-purulent conjunctivitis. Clinical symptoms appear sequentially and there are no specific laboratory tests for KD; thus, diagnosing KD with atypical presentation is challenging for clinicians, resulting in detrimental therapeutic delays. Due to prolonged fever and elevated inflammatory markers, both patients were initially misdiagnosed with sepsis and treated with antibiotics without improvement. Owing to the emergence of major clinical criteria for KD, definitive diagnosis was done upon admission to our hospital.

KD is the leading cause of acquired heart disease in children. Cardiac diseases are the most common and serious complications of KD, which include coronary artery aneurysm, myocarditis, valvular abnormalities, and endothelial dysfunction (10). The incidence of CAAs is approximately 25% among patients who remain untreated. KD patients with systolic hypotension or shock are known to have KDSS. Severe hypotension in KD may be related to proinflammatory cytokine overexpression, myocardial dysfunction, and vasculitis with ongoing capillary leakage (11). KDSS was defined as KD with either clinical manifestations of poor perfusion regardless of measured blood pressure or > 20% decrease in systolic blood pressure (4). Both patients met the criteria for KDSS.

MAS is another life-threatening complication of multiple systemic inflammatory diseases, and is most commonly seen in systemic juvenile idiopathic arthritis (sJIA) but less commonly in KD. The hallmark of MAS is a cytokine storm caused by excessive cytokine production, haemophagocytosis, and monocyte/macrophage activation. Currently, there is still no standard criterion for MAS in KD. The most widely used criteria for the diagnosis of MAS are still the HLH criteria or criteria for MAS in sJIA, such as the HLH 2004 criteria and 2016 sJIA-MAS criteria. Although the HLH-2004 criteria, which were originally developed for primary HLH, are useful, they are inappropriate for diagnosing MAS in secondary HLH, including KD. Hemophagocytosis is a characteristic feature of MAS as part of the HLH-2004 criteria; however, this finding may not be present, particularly in the early stages (6). Splenomegaly is also included in the HLH-2004 criteria and considered a potential clinical clue of KD complicated with MAS (6, 12). Both cases had no hemophagocytosis and splenomegaly. Furthermore, both soluble CD25 levels and natural killer cell function, which are included in the HLH-2004 criteria, are not routinely examined in most hospitals. According to the 2004 HLH diagnostic criteria, both patients in our study did not have MAS, which may have resulted in delayed treatment. However, the parameters of 2016 sJIA-MAS criteria can be measured in blood at relatively low cost and wide availability; hence, these are useful for MAS screening. Moreover, the 2016 sJIA-MAS criteria are recommended for detecting KD complicated with MAS (13), which are highly sensitive and specific compared to the HLH-2004 criteria (6). In this study, both patients met the 2016 sJIA-MAS diagnostic criteria.

Gastrointestinal symptoms are relatively common in KD, including vomiting, diarrhea, abdominal pain, and abdominal distension (14). It has been reported that up to 91% of KDSS patients have gastrointestinal manifestations (15), which may be related to a more intense systemic vasculitis. Nonetheless, acute surgical abdomen is rare in patients with KD. At an earlier stage, gastrointestinal symptoms may occur before the onset of all typical symptoms of KD, resulting in the misdiagnosis of surgical diseases, as seen in case 1. Meanwhile, most patients with KD complicated by acute abdomen often undergo unnecessary surgery without improvement due to insufficient recognition of this disease (16–18). Severe cases may cause obstruction or even perforation, which should require prompt surgical intervention. Intestinal perforation in KD, as in case 2, is rare, and only two cases have been reported to date (18, 19).

During the COVID-19 pandemic, a novel immune-mediated hyperinflammatory condition associated with SARS-CoV-2 infection or exposure was termed multisystem inflammatory syndrome in children (MIS-C) and firstly reported in April 2020 in the United Kingdom. MIS-C had characteristics overlapping with KD, KDSS, and MAS (20–23). Both cases showed symptoms of fever, skin rash, conjunctivitis, and oral mucosal changes, which could be found in KD and MIS-C. And several atypical features remind us to consider MIS-C in the differential diagnosis, such as older age, gastrointestinal symptoms, rapidly progressing shock, hematological findings including neutrophilia, hyponatremia, and hypoalbuminemia. However, KDSS and KD-MAS are also usually seen in children older than 5 years old (6, 10). Gastrointestinal symptoms were common in KDSS. The key features of MAS, including hypofibrinogenemia, splenomegaly, hypertriglyceridemia and bone marrow hemophagocytosis, are not usually described in MIS-C (21). The serology test and RT-PCR for SARS-CoV-2 were negative in both cases. Positive results of SARS-CoV-2 are low by RT-PCR but high by serological testing, indicating past infection. In addition, the two cases had no exposure history and people were not affected by COVID-19 after contact with the both patients. Meanwhile, Mianyang remained a low incidence area in 2020. Hence, KD with KDSS and MAS but not MIS-C was diagnosed in our cases.

KDSS, MAS and severe acute abdomen are life-threatening complications of KD, which require more aggressive treatment. The standard KD therapy with IVIG and aspirin is fundamental. However, these patients tend to have a high risk of IVIG resistance and CAAs (5, 8, 9), requiring additional IVIG. The patient in case 2 experienced IVIG resistance and coronary dilation; thus, a second IVIG was administered. As KDSS is characterized by shock, anti-shock therapy, such as fluid resuscitation and vasoactive drugs, is critical. Hyperinflammation due to cytokines storm is a hallmark of these conditions; hence, potent anti-inflammatory medications are needed. The administration of high doses of corticosteroids is considered the initial treatment. If not effective, adjunctive therapy with immunomodulatory and biological agents, such as infliximab, anakinra, or cyclosporine, can be considered (24–26). In the present study, both patients received high-dose intravenous methylprednisolone pulse therapy with improvement. Moreover, comprehensive treatments are also required based on the disease progression, as these patients are usually critically ill.

In conclusion, this case report describes an unusual combination of KDSS, MAS, and acute abdomen in KD patients. It is important to consider KD in children with fever, unexplained gastrointestinal symptoms, and one or two of the major clinical signs of KD. KDSS and MAS are occasionally present simultaneously. Timely diagnosis and treatment are vital as they can avoid surgery in a proportion of cases and prevent complications.

## Data Availability

The original contributions presented in the study are included in the article/Supplementary Material, further inquiries can be directed to the corresponding authors.
